# A giant cerebriform submucosal lipoma extending from the gastric body to the duodenal bulb: a case report

**DOI:** 10.1055/a-2621-3392

**Published:** 2025-07-02

**Authors:** Tian-Xing Yuan, Ye-Han Zhou, Yu Bao, Rui Zhao

**Affiliations:** 1 12599 School of Medicine, University of Electronic Science and Technology of China, Chengdu, China; 2 Department of Pathology, Sichuan Cancer Hospital and Institute, Chengdu, China; 3 Department of Endoscopy, Sichuan Cancer Hospital and Institute, Chengdu, China

Lipomas are benign tumors composed of mature adipocytes. Gastrointestinal (GI) lipomas are rare neoplasms, particularly those occurring in the stomach, which account for only 5% of all GI lipomas and 2%–3% of all benign gastric tumors. They are most commonly found in the gastric antrum 1 2 . Gastric lipomas are typically small and asymptomatic; however, as they enlarge, symptoms such as epigastric discomfort, gastric outlet obstruction, and dyspepsia may occur 3 .

We report the case of a 47-year-old man who was referred to our department following the detection of a large submucosal protrusion during an upper GI endoscopy at an external hospital. Esophagogastroduodenoscopy revealed a giant subepithelial lesion ( Fig. 1 ). Extensive, thickened, fold-like and nodular mucosal elevations resembling cerebriform changes were observed from the greater curvature of the lower gastric body to the antrum. A prominent protrusion extended into the duodenal bulb through the pylorus, with smooth overlying mucosa and a soft texture. Endoscopic ultrasonography demonstrated that the lesion was primarily located in the submucosa, with localized thickening up to 1.6 cm, exhibiting homogeneous hyperechogenicity. Elastography indicated a soft consistency, consistent with the characteristics of a lipoma. Contrast-enhanced computed tomography revealed nodular and mass-like lesions with fat density in the gastric body, antrum, and adjacent duodenal region, with the largest measuring approximately 7.2 × 5.8 cm, suggestive of a lipoma ( Fig. 2 ).

**Fig. 1:**
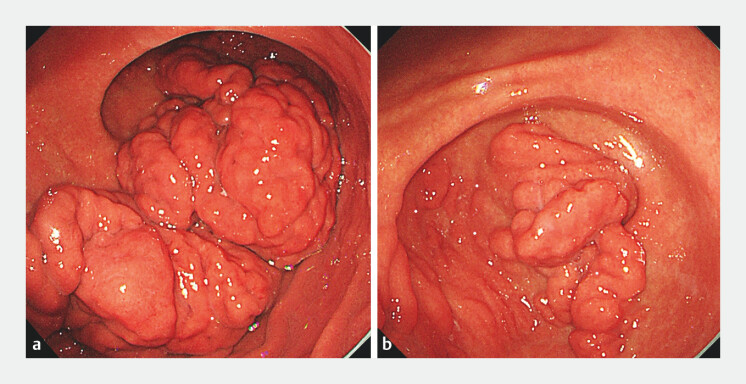
Endoscopic findings. **a** Giant cerebriform submucosal lipoma in the gastric body. **b** Giant cerebriform submucosal lipoma in the gastric antrum.

**Fig. 2:**
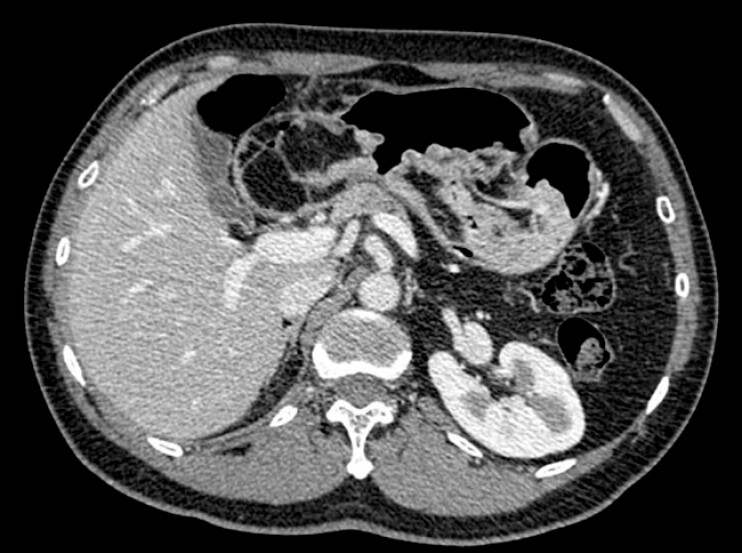
Contrast-enhanced computed tomography showed fat-dense nodules.

Endoscopic snare electrocautery resection was performed to remove two large tissue specimens for pathological examination, with no postoperative bleeding observed at the resection site ( Video 1 ).

A giant cerebriform submucosal lipoma extending from the gastric body to the duodenal bulb.Video 1

Histopathological examination of the biopsy specimens confirmed the presence of mature adipose tissue in the lamina propria and submucosa, supporting the diagnosis of a lipoma ( Fig. 3 ).

**Fig. 3:**
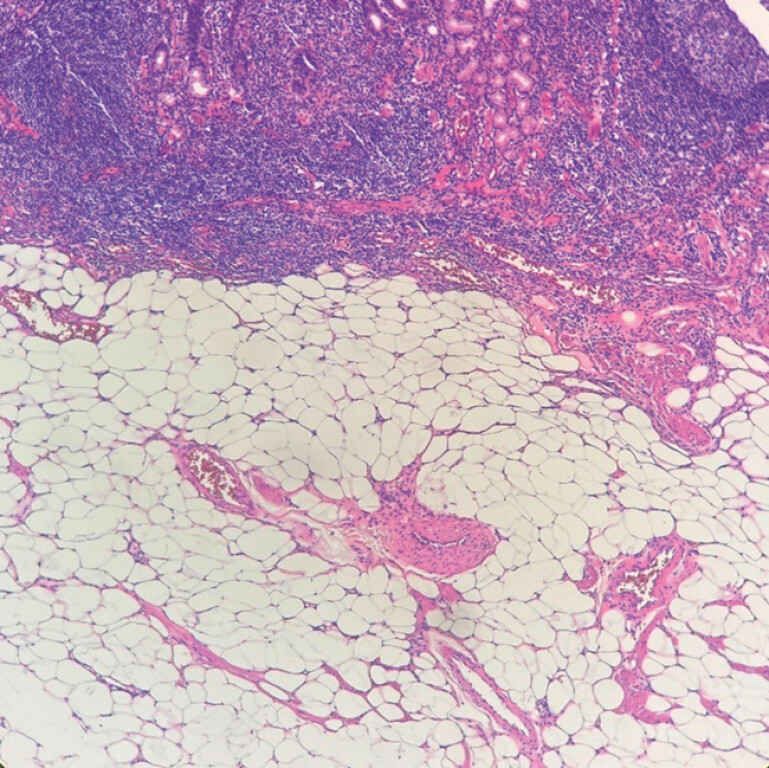
Histological analysis revealed mature fat tissue in the lamina propria and submucosa, diagnostic of lipoma (hematoxylin and eosin ×100).

To our knowledge, this is the first reported case of a giant gastric lipoma exhibiting cerebriform morphology and extending from the gastric body to the duodenal bulb. Although the lesion was extensive, the intact mucosa and absence of malignant features supported a benign diagnosis. Conservative management with regular follow-up may be considered; however, endoscopic resection should be performed if symptoms such as obstruction, bleeding, or ulceration develop 4 .

Endoscopy_UCTN_Code_CCL_1AB_2AD_3AB

## Notes

Endoscopy E-Videos https://eref.thieme.de/e-videos
*E-Videos* is an open access online section of the journal *Endoscopy* , reporting on interesting cases and new techniques in gastroenterological endoscopy. All papers include a high-quality video and are published with a Creative Commons CC-BY license. Endoscopy E-Videos qualify for HINARI discounts and waivers and eligibility is automatically checked during the submission process. We grant 100% waivers to articles whose corresponding authors are based in Group A countries and 50% waivers to those who are based in Group B countries as classified by Research4Life (see: https://www.research4life.org/access/eligibility/ ). This section has its own submission website at https://mc.manuscriptcentral.com/e-videos .

## References

[1] Cappell MS Stevens CE Amin M Systematic review of giant gastric lipomas reported since 1980 and report of two new cases in a review of 117110 esophagogastroduodenoscopies World J Gastroenterol 2017 23 5619 28852321 10.3748/wjg.v23.i30.5619 PMC5558125

[2] Sullivan IW Hota P Dass C Gastric lipomas: a case series and review of a rare tumor BJR Case Rep 2019 5 2.0180109E7 10.1259/bjrcr.20180109 PMC6726183 31501708

[3] Krasniqi AS Hoxha FT Bicaj BX Symptomatic subserosal gastric lipoma successfully treated with enucleation World J Gastroenterol 2008 14 5930 18855998 10.3748/wjg.14.5930 PMC2751909

[4] Deprez PH Moons LMG OʼToole D Endoscopic management of subepithelial lesions including neuroendocrine neoplasms: European Society of Gastrointestinal Endoscopy (ESGE) Guideline Endoscopy 2022 54 412 429 10.1055/a-1751-5742 35180797

